# Medical Signal Processing in Biomedical and Clinical Applications

**DOI:** 10.1155/2018/3932471

**Published:** 2018-06-12

**Authors:** Kunal Pal, Kunal Mitra, Arindam Bit, Saugat Bhattacharyya, Anilesh Dey

**Affiliations:** ^1^Department of Biotechnology and Medical Engineering, National Institute of Technology, Rourkela, India; ^2^Department of Biomedical Engineering, Florida Institute of Technology, Melbourne, FL, USA; ^3^Department of Biomedical Engineering, National Institute of Technology, Raipur, India; ^4^School of Computer Science and Electronic Engineering, University of Essex, Colchester, UK; ^5^Department of Electronics and Communication Engineering, Kaziranga University, Jorhat, India

Biological signals are the reflection of accumulated action potentials of subdermal tissues of a living being. Its presence signifies the ionic and electrical activities of the muscular and the neural cells in a synchronized manner. Being a mosaic model of a living architecture, the resultant vectors of biological signals have temporal as well as spatial representation. These signals are stochastic in nature. Medical diagnostic tools are prevalent using the support of medical signals. In the course of time, a significant amount of progress has been achieved in the field of medical signal processing for the improvement of the signal-to-noise ratio, extraction of features from those filtered signals, and classification of the extracted signals for clinical applications. This special issue emphasized the recent development of medical signal processing, improvement of algorithms, and wider clinical applications. Entropy-based kernel extraction technique is being used for the analysis of the nonlinear and nonstationary epoch signals. This kind of approach shows robustness in noise reduction. Machine learning algorithms are also being used for real-time feature extraction (pattern extraction) from tympanic temperature profiles. Quadratic support vector machine algorithms were also found to enhance the accuracy of the detection mechanism.

Regulation of rehabilitation devices and protocols are also governed by the processing of medical signals. Reference techniques are often used for acquiring surface EMG signals for the activation of the rehabilitation actuators. Consecutive placement of stimulator-detector arrays influences the spatial acquisition of the functional electrical stimulus (FES) and volitional sEMG which can be used for controlling EMG-driven FES neuroprosthesis. Dynamic models are also being used for analyzing and detecting features from sEMG of deltoid muscles, present in the upper arm. The analysis of the signals can be used for the evaluation of the differently abled persons to predict the treatment outcomes. Health monitoring can also be achieved by processing of time-domain biological signals of sub-nanosecond durations. It can even help in predicting the state of the human heart by correlating the activity of the heart muscles and the blood pumping process.

Deep recurrent neural networks are also found to be useful tools for predicting features related to the adverse effect of drugs on the human body. In addition to this technique, conditional random fields are also implemented by many researchers for identifying the biological signals from significantly highly correlated background noise due to the subsequent physiological modification after the introduction of the foreign particles within the body. Similarly, the assessment of the trace elements in the human body can also be achieved by extracting features of the biological signals and biomarkers, and subsequently analyzing the features. A neural network can play an important role towards the identification of the continuously adaptable trace elements within the physiological fluids.

Biomedical signaling has significant outreach in its clinical application domain. Cerebral arterial stenoses can also be predicted by a nonradioactive technique, namely, photoplethysmography. It involves the employment of an optical detection technique, which is a wavelength-specific process. Corresponding photodetectors are being used to measure the wavelength of the reflected light. Cerebral disorder can also be detected from EEG-fMRI recordings. It involves a rigorous filtering process, which employs a comb filter, followed by a moving-averaged filter for the elimination of the random noises.

In this special issue, all the above topics are discussed with their recent state of the art and their corresponding clinical applications.

*Kunal Pal* *Kunal Mitra* *Arindam Bit* *Saugat Bhattacharyya* *Anilesh Dey*

